# Effects of the dementia care toolbox on personnel’s self-reported confidence in patient care: a CRT in general practices

**DOI:** 10.1186/s12875-021-01577-8

**Published:** 2021-11-17

**Authors:** Sabine Christine Jäger, Anna-Liesa Filbert, Thomas Welchowski, Birgitta Weltermann

**Affiliations:** 1 Institute of General Practice and Family Medicine, University Hospital Bonn, University of Bonn, Venusberg-Campus 1, 53127 Bonn, Germany; 2 Institute of Medical Biometry, Informatics and Epidemiology, University Hospital Bonn, University of Bonn, Bonn, Germany

**Keywords:** Dementia care, Dementia, General practitioners, Self-reported confidence in primary care, Intervention, Migration background

## Abstract

**Background:**

In rapidly aging populations, general practitioners (GPs) are challenged in dementia care of patients with and without migration background. Uncertainties in treating dementia in migrant patients due to language barriers or information deficits are reported. To address these deficits, we developed the Dementia Care Toolbox which was judged helpful by GP practice personnel. This two-armed cluster-randomised trial (CRT) investigated the effects of this toolbox on German GPs’ and practice assistants’ (PrAs) attitudes and confidence in dementia care, especially in patients with migration background.

**Methods:**

A total of 32 GP practices were recruited and randomised into intervention (toolbox use for 3 months) and waiting-list control (toolbox after follow-up). After 3 months all participating GPs and PrAs received a standardised questionnaire addressing their levels of self-reported confidence in dementia care for patients with and without migration background. A generalized estimating equation model that took practice cluster effects into account was applied to assess GPs and PrAs self-reported confidence in dementia care in patients with and without migration background.

**Results:**

Overall, the intervention had no significant effect on self-reported confidence in dementia care. However, the use of the dementia care toolbox showed a tendency for a learning effect on knowledge about local support structures for migrant patients with dementia (odds ratio 1.43; 95% CI 0.68-3.03, *p* = 0.35) and for less communication difficulties with dementia patients in general (odds ratio 0.72; 95% CI 0.33-1.56; *p* = 0.40). Moreover, intervention practices showed a tendency towards more awareness of own limitations: less self-confidence regarding answering questions (odds ratio 0.82; 95% CI 0.36-1.86, *p* = 0.64) and providing information for patients with migration background (odds ratio 0.60; 95% CI 0.25-1.45, *p* = 0.26).

**Conclusion:**

The Toolbox Dementia Care increased awareness on the respective topic. Given a small sample size, further studies on its effectiveness in primary care are needed.

**Trial registration:**

German Clinical Trials Register, DRKS00014632. Registered 02/08/2018.

## Background

Dementia is a worldwide challenge due to a lack of curative therapies for most forms of the syndrome [1]. It is estimated that 46.8 million people suffer from dementia and this number is expected to triple by 2050 [2]. Especially in aging populations, this represents a considerable economic, medical, and social challenge for health care systems [3–6]. Although an issue of debate [1], it is believed that the (early) detection of dementia benefits the patient and their next of kin in terms of optimal treatment, reduction of psychological stress and the possibility of living in a familiar environment as long as possible [7–10]. British studies from primary care by Cahill et al. [11] and Iliffe et al. [12] showed that detection can be challenging as early symptoms are difficult to distinguish from those of other cognitive impairments and normal ageing processes, testing is time-consuming [13, 14] and diagnostic skills may be insufficient [14, 15]. Surveys among general practitioners (GPs) from Ireland [11, 16], Nepal [15] and England [17] indicate a lack of knowledge on dementia [18] and support services available for patients and their next of kin [14, 19], as well as uncertainties in communicating the diagnosis [14, 18], especially when dealing with migrant populations [20]. The authors of these studies recommended information and support strategies for both physicians as well as patients and their care givers [14, 18, 11].

In Germany, 21.2 million inhabitants have a migration background of whom 2.07 million are aged over 65 years and are at risk for dementia [21]. European studies from Nielsen et al. indicate that diagnosing dementia in migrants is considered difficult by two thirds of physicians [20]. Therefore, researchers from Belgium and Norway assumed that dementia is underdiagnosed in migrants [22, 23]. Currently, there is no data from Germany indicating an outcome-relevant deficit in dementia care for migrants, but this may differ from other countries as all patients have health coverage and access to primary and further level neuro-psychiatric care. For medical and ethical reasons, the German guideline on dementia recommends dementia diagnostic only in patients agreeing on this [1]. Aiming to better understand potential difficulties of German GPs in dementia care for patients with and without migration background, we had performed a physician questionnaire survey among 326 GPs: 96% experienced barriers at least once mostly due to language barriers or information deficits for migrants, 70.9% reported difficulties in diagnosing dementia in migrants [24]. To provide focussed information for GP practices, we developed a dementia care toolbox with material for physicians and practice assistants (PrAs) (information card, diagnostic tool). In addition, information media for patients and their next of kin (poster for the waiting room, brochure) was provided in German, Turkish and Russian. This is in accordance with the distribution of migrants in the German North Rhine region, where most migrants have a Turkish (17.7%) or Russian (8.1%) background [25]. The effects of the toolbox on GPs self-reported care for dementia care patients with and without migration background was studied in the intervention arm of this study [26]. In a first analysis, we showed that the toolbox was generally well accepted (82%) by GPs and PrAs. In descending order, both professions rated the brochures (52.1.%), the information card (44.9%) and the poster (28.6%) as helpful [26]. Here we report the effects of the toolbox on the self-reported confidence of German GPs and PrAs when dealing with dementia patients with and without a migration background.

## Methods

### Study design and participants

This two-armed cluster-randomised trial (CRT) targeted GPs and PrAs in the German North Rhine region. The intervention comprised the Dementia Care Toolbox for GP practices [26]. Details of the study protocol for the cross-sectional study are published [27]; the study protocol for this CRT was submitted to the Ethics Committee of the Medical Faculty of the University of Bonn and is detailed in this manuscript. In short, a total of 320 general practices were invited to participate: 1) 230 GPs from the random sample of the prior cross-sectional study and 2) 90 practices from the institute’s teaching practice network with known research interested were asked for participation in the study [24]. A total of 46 practices participated either in the intervention or control group with follow-up data available for 32 practices [26]. Due to the novelty of the intervention, sample size was estimated based on prior CRTs in general practices. Practices were allocated to intervention or wait list-controlled group by random number generator. Intervention practices received the Dementia Care Toolbox by mail after randomisation. Practices in the waiting list-control arm received the intervention after the follow-up data collection was completed. Scientists involved in the project were responsible for study conduct, including randomisation, enrolment of participants and assignment to intervention groups. No blinding was performed. The study took place from September 2018 to April 2019, with the intervention being conducted from September to November 2018.

### Intervention: description of dementia care toolbox

All practices who provided written informed consent were randomised and received the Dementia Care Toolbox by mail with the offer to use the materials for the subsequent 3 months. After 3 months, physicians and PrAs received a questionnaire to assess for the use in the toolbox [26] and effects on self-reported confidence in patient care.

The Dementia Care Toolbox comprised the following material:

1. Patients and next of kin
  ◦ Brochures: The 8-page brochure gave an overview on dementia and included a definition of dementia and core symptoms, contact addresses of support services as well as the further procedure after first dementia symptoms appeared. It was available in German, Turkish and Russian.
  ◦ Poster (30 cm × 42 cm): The poster was hung up in the waiting room of the practice to raise awareness of dementia among patients and next of kin. Short questions and statements about core symptoms of dementia were printed on the poster in German, Turkish and Russian.
2. GPs and PrAs:

- ◦ Information card: The double-sided information card contained general information on dementia as well as assistance for challenges, communication, cultural sensitivity for dementia patients with and without a migration background and contact addresses in case of language barriers.

3 GPs

- ◦ Practical tool: To facilitate the diagnostic of dementia in patients with and without a migration background, the practical tool included double-sided printed medical history sheets in Turkish/German, English/German and Russian/German, a non-verbal, culturally sensitive screening test for cognitive impairment and a 20-page booklet on dementia.

Details of the intervention material are published [26] and shown in Table 1.

**Table 1:** Description of the intervention toolbox

Target group	Item	Aim of the material	Topic/Content	Layout	Language
Patients, next of kin	8-page brochure	Provide overview and support	• Definition of dementia and symptoms • Contact addresses • Procedural steps (GPs)	• Symbols • Diagram • Pictures • Highlighted keywords • Websites	Common language: German Russian Turkish
Patients, next of kin	Poster (30 cm × 42 cm)	Creating awareness	• Questions about key symptoms of dementia	• Symbols	Common language: German Russian Turkish
GP, PrA	2-page information card	Information on how to deal with patients with and without a migration background	• Support services for language barriers • Cultural sensitivity • Contact addresses • Communication advice	• Symbols • Highlighted keywords • Websites	German
GP	Practical tool	Facilitation of diagnostics for people with and without a migration background	• Medical history sheet • EASY test • 20-page booklet	• Highlighted keywords • Symbols • Interviews • Diagrams • Websites	German-English German-Turkish German-Russian

Target group: *GP* General practitioner, *PrA* Practice assistant

### Questionnaires

GPs and PrAs were asked to fill a standardised, self-administered questionnaire after 3 months. In addition to sociodemographic data (age, gender, migration background), the level of agreement with the following six statements on self-reported confidence in dementia care were requested. Answer options ranged from “totally disagree” to “totally agree” on a 5-point Likert scale:

- I feel confident in dealing with dementia patients with migration background.
- I feel confident to inform dementia patients with migration background about their disease.
- I feel confident in answering question from dementia patients with migration background.
- I have enough knowledge about local help centres that support dementia patients with a migration background and their families.
- I often have difficulties communicating with patients with migration background.
- I often have difficulties communicating with dementia patients.

Practices were reminded once by mail and twice by phone. An access limited masterfile contained the name of the physician and contact data of the practice, which was used for pseudonymization. The names of the participating PrAs were not requested. Each GP had provided the number of participating PrAs and a respective number of questionnaires for these team members.

### Statistical methods

Questionnaires had been read in with TeleForm data capture system with subsequent visual control. Relative frequencies, means and quartiles were used to describe the characteristics of the intervention and control arm. Chi-square tests were used to compare the categorical variables such as gender, occupation, and migration background between both groups, while the t-test was applied for continuous variables. i.e., age and working years. Generalized Estimating Equation (GEE) models [28] that took practice cluster effects into account were applied to examine effects of the Dementia Care Toolbox on GPs’ and PrAs’ levels of self-reported confidence in dementia care of patients with and without migration background (primary outcome). Responses on the 5-point Likert scale were merged into 3 categories: fully disagree/ disagree, neutral and agree/fully agree. The GEE model was run with a first order autoregressive relationship (AR-1) working correlation matrix including the factors condition, occupation, gender, and migration background to determine the overall effects on self-reported confidence in dementia care (all six items) with a Poisson distribution (log link) and each item separately with a multinomial distribution (logit link). Effects were assessed on the base of Odds Ratios (OR) with a 95% confidence interval (CI). IBM SPSS Statistics, Version 26, was used for statistical analysis with a significance level set at *p* = 0.05.

### Ethics statement

The study was approved by the Ethics´ Committee of the Medical Faculty of the University of Bonn. It was registered in the German Clinical Trials Register on 02/08/2018 (DRKS-ID: DRKS00014632) [29].

## Results

### Participants’ characteristics

The intervention group comprised 50 participants, of whom 14 were GPs and 36 PrAs whereas the control group consisted of 16 GPs and 57 PrAs. Most respondents had no migration background (intervention: 85.4%, control 83.6%). The majority of physicians were male (intervention: 71.4%, control: 56.3%), while the majority of PrAs were female (intervention: 97.2%, control: 100%). The mean age of the participants in the control group was 44.97 years (SD ± 11.71) and in the intervention group 45.44 years (SD ± 13.70). After dichotomizing age into < 50 years and > =50 years, the distribution of the intervention group was balanced (< 50 years: 50%, > = 50 years: 50%), whereas 58.3% participants of the control group were aged < 50 years. Most GPs and PrAs of both groups worked in their general practice for more than 15 years (intervention: 46.9%, control: 40.3%). Both groups did not show any significant difference in age (*p* = 0.10), working years (*p* = 0.41), sex (*p* = 0.15), occupation (*p* = 0.44) and migration background (*p* = 0.78). The practice teams comprised one to eight participants (at least one GP per practice with differing numbers of PrAs). For details see Table 2 and [26].

**Table 2:** Characteristics of the study participants

	Intervention group		Control group	
	**n**	**(%)**^**a**^	**n**	**(%)**^**a**^
**Total participants**	50	(40.6)	73	(59.4)
**Total practices**	15	(46.9)	17	(53.1)
	**n**	**(%)**^**a,b**^	**n**	**(%)**^**a,b**^
**Profession**				
GPs	14	(28.0)	16	(21.9)
*Male*	10	(71.4)	9	(56.3)
*Female*	4	(28.6)	7	(43.8)
PrAs	36	(72.0)	57	(78.1)
*Male*	1	(2.8)	0	(0)
*Female*	35	(97.2)	57	(100.0)
**Gender**				
Male	11	(22.0)	9	(12.3)
Female	39	(78.0)	64	(87.7)
**Was your mother or father or were you yourself born abroad?**				
Yes	7	(14.6)	12	(16.4)
No	41	(85.4)	61	(83.6)
**Age**				
< 50	25	(50.0)	42	(58.3)
> =50	25	(50.0)	30	(41.7)
**Duration of employment in this family practice**				
< =5 years	12	(24.5)	17	(23.6)
< =15 years	14	(28.6)	26	(36.1)
> 15 years	23	(46.9)	29	(40.3)

^a^ Column percentages

^b^ Percentages reported for valid cases

Regarding levels of self-reported confidence in dementia care most GPs and PrAs in both groups agreed with one of the six statements (intervention: 27.1%, control: 31.9%), followed by agreeing with none (intervention: 22.9%, control: 23.6%) and with two items (intervention: 22.9%, control: 19.4%). Particularly, more participants in the intervention group did not agree on feeling confident in dealing with (intervention: 50.0%, control: 43.1%) or providing information about dementia to migrants compared to the control group (intervention: 60.4%, control: 52.8%). In contrast, more respondents of the control group indicated a lack of knowledge about local support opportunities for migrants with dementia and their families compared to the intervention (intervention: 54.2%, control: 61.1%). For details see Tables 3 and 4. In the intervention group, 57.4% of the participants rated the communication with dementia patients in general as uncomplicated, as did 47.2% of the control group. Communication with dementia patients with migration background was rated either as difficult (intervention: 44.7%, control: 30.6%) or non-problematic in equal proportions (intervention: 42.6%, control: 30.6%). For details see Table 4.

**Table 3:** Overall self-reported confidence in dementia care: frequency of answer options ‘agree/ fully agree’ per participant

	Intervention group		Control group	
	n	(%)^**a,b**^	n	(%)^**a,b**^
**0**	11	(22.9)	17	(23.6)
**1**	13	(27.1)	23	(31.9)
**2**	11	(22.9)	14	(19.4)
**3**	7	(14.6)	9	(12.5)
**4**	3	(6.3)	5	(6.9)
**5**	3	(6.3)	4	(5.6)
**6**	0	(0)	0	(0)
**Mean ± SD**	1.73 ± 1.46		1.64 ± 1.44	

^a^ Column percentages

^b^ Percentages reported for valid cases

**Table 4:** Frequencies of self-reported confidence in dementia care for all six questions separately

	Intervention group		Control group	
	n	(%)^**a,b**^	n	(%)^**a,b**^
**I feel confident in dealing with dementia patients with migration background**				
(Fully) Disagree	24	(50.0)	31	(43.1)
Neutral	19	(39.6)	28	(38.9)
(Fully) Agree	5	(10.4)	13	(18.1)
**I feel confident to inform dementia patients with migration background about their disease**				
(Fully) Disagree	29	(60.4)	38	(52.8)
Neutral	9	(18.8)	18	(25.0)
(Fully) Agree	10	(20.8)	16	(22.2)
**I feel confident in answering question from dementia patients with migration background**				
(Fully) Disagree	19	(39.6)	28	(38.9)
Neutral	17	(35.4)	23	(31.9)
(Fully) Agree	12	(25.0)	21	(29.2)
**I have enough knowledge about local help centres that support dementia patients with a migration background and their families**				
(Fully) Disagree	26	(54.2)	44	(61.1)
Neutral	13	(27.1)	16	(22.2)
(Fully) Agree	9	(18.8)	12	(16.7)
**I often have difficulties communicating with patients with migration background**				
(Fully) Disagree	20	(42.6)	22	(30.6)
Neutral	6	(12.8)	28	(38.9)
(Fully) Agree	21	(44.7)	22	(30.6)
**I often have difficulties communicating with dementia patients**				
(Fully) Disagree	27	(57.4)	34	(47.2)
Neutral	14	(29.8)	26	(36.1)
(Fully) Agree	6	(12.8)	12	(16.7)

^a^ Column percentages

^b^ Percentages reported for valid cases

### Effects of the dementia care toolbox on self-reported confidence in dementia care

The intervention Dementia Care Toolbox had no significant effects on self-reported confidence in dementia care, neither on the single six aspects addressed nor the summarizing item (*p* = 0.95; Table 5). However, a tendency was found among the intervention group who showed a learning effect in terms of increased knowledge about local support options for migrant patients and their families compared to the control group (OR = 1.43; 95% CI = 0.68-3.03, *p* = 0.35). In addition, intervention practices were more likely to experience less communication difficulties with dementia patients in general (OR = 0.72; 95% CI = 0.33-1.56, *p* = 0.40). In comparison to these learning effects, practices of the intervention group showed a tendency of feeling less confident in answering questions of migrants with dementia compared to the control group (OR = 0.82; 95% CI = 0.36-1.86, *p* = 0.64). Both groups also differed in terms of the fact that the intervention group showed a tendency to experience more communication difficulties with patients with a migration background (OR = 1.63; 95% CI = 0.65-4.10, *p* = 0.30). In addition, they showed a higher chance of feeling less confident to inform this group of patients sufficiently about dementia (OR = 0.60; 95% CI = 0.25-1.45, *p* = 0.26) and to deal with them (OR = 0.57; 95% CI = 0.26-1.23, *p* = 0.15), For details see Table 5.

**Table 5:** Results GEE model on the overall effects and all six aspects separately on self-reported confidence

Item	Sig	OR	95% CI
Overall effect on self-reported confidence in dementia care.	0.95	1.01	0.72-1.43
I feel confident in dealing with dementia patients with migration background.	0.15	0.57	0.26-1.23
I feel confident to inform dementia patients with migration background about their disease.	0.26	0.60	0.25-1.45
I feel confident in answering question from dementia patients with migration background.	0.64	0.82	0.36-1.86
I have enough knowledge about local help centres that support dementia patients with a migrant background and their families.	0.35	1.43	0.68-3.03
I often have difficulties communicating with patients with migration background.	0.30	1.63	0.65-4.10
I often have difficulties communicating with dementia patients.	0.40	0.72	0.33-1.56

*Control group* reference; controlled for occupation (GPs, PrAs), sex (male, female), migration background (yes, no)

## Discussion

This cluster-randomised controlled intervention trial aimed at raising awareness and perceived self-confidence on dementia care for patients with and without migration background in German GPs and PrAs by providing the newly developed Dementia Care Toolbox. Based on a sample with 32 GP practices only, our toolbox did not show a significant effect on self-reported confidence in dementia care after 3 months, yet there were positive tendencies indicating learning effects regarding knowledge on local support centres and self-reported communication skills with dementia patients. The latter findings are in line with previous studies stressing an educational need for increasing GPs´ self-confidence in dementia care [11, 18]. Several studies from various countries addressed this problem and studied interventions aiming to improve dementia care in primary care. In a large, nationwide dementia-focused Continuing Medical Education program with 1352 GPs from Australia, Casey et al. 2020 found a significant increase in dementia awareness and self-reported confidence directly after the intervention and at 6 to 9 months follow-up measured with a self-report survey using mainly Likert scales [30]. In comparison to our 3-months intervention with printed materials only, the former-mentioned program offered at least 6 h online or face-to-face education including case studies and discussions to GPs. Likewise, 29 American primary care physicians, who participated in a one-day dementia care training program, showed a significantly increase in dementia care competency after 6 months assessed with a baseline, post-training, and follow-up questionnaire [31]. Similar improvements of interventions in dementia care were shown in some European GP populations [32]. Foley et al. conducted a study with 104 GPs from Ireland who participated in small-group dementia workshops resulting in increased knowledge and confidence in dementia care measured with post-intervention questionnaires using Likert-style response options [32]. As questionnaires of these studies differed, comparisons on dementia related attitudes and self-reported confidence need to be considered with caution. Our study used the validated and reliable General Practitioner Attitudes and Confidence Scale for Dementia (GPACS-D) developed by Mason et al. for measuring Australian GPs attitudes and confidence in dementia care [33]. In contrast to these studies, our study is the first survey targeting GP practices´ dementia care in patients with migration background. Although no significant differences were found in the limited sample, we showed tendencies of reduced self-reported confidence in dementia care of patients with migration background in the intervention group. This might suggest that our intervention “dementia care toolbox” might have increased GPs´ and PrAs´ awareness of their limitations in care for patients with migration background. Reported difficulties and challenges of GPs addressed communication with and information of dementia patients with migration background which highlights the need for special interventions. Similar difficulties in dementia care for patients with migration background were shown in previous studies. In a study of 27 health professionals from Norway, Sagbakken et al. (2018) found that language barriers and a lack of appropriate diagnostic tools represented main barriers for GPs in dementia care for migrants [34]. These authors recommended that communication and diagnostic skills should be improved by offering educational trainings to GPs. Similar conclusions were drawn from a Swiss survey among 4460 GPs of whom 15% reported a lack of confidence in diagnosing dementia in patients with a migration background [35]. Likewise, a survey of 36 clinical dementia centres from 15 European countries showed that 64% reported difficulties in diagnosing dementia in migrants due to communication problems and insufficient diagnostic tools [20]. Our toolbox addressed these deficits by offering practical tools for GPs in several languages. In addition, the toolbox included information brochures for patients and next of kin in several languages as families play a major role in early dementia detection. Such an approach is supported by a 2020 published study from Norway addressing barriers and facilitators for dementia care service in eight family caregivers of migrants with dementia [36]: a lack of knowledge on dementia and insufficient awareness of care services for migrants with dementia were identified as key barriers. Also, intercultural differences need to be respected as dementia is not socially accepted as a neurological disease in all cultures and initial symptoms may be misinterpreted as normal aging [37].

## Strengths and limitations

This cluster-randomised study addressed both professional groups involved in GP practices´ care for dementia patients, namely GPs and PrAs which is a major strength of our study. Due to limited project resources, this exploratory study has several limitations. First, no studies with reliable outcomes addressing knowledge and confidence of German GPs in dementia care for migrants were available for a formal sample size calculation. The focus was a comparison of the toolbox effects between intervention and control arm at follow-up as this information was not assessed at baseline. Second, no qualitative information on changes in practices and the use of the toolbox was obtained. Third, the intervention might have drawn attention to general practices with particular interest in dementia care, so that results on self-reported confidence in dementia care might not be representative for all GPs. German GPs complete at least 5 years of postgraduate training prior to licensing, while GP training comprises 3 years in many other countries. Therefore, generalizability of the present findings might be limited. Fourth, our questionnaire was a modified version of the Australian GPACS-D which is not validated in German yet. As we showed a high acceptance of the toolbox materials in our prior publication [26], a larger study is reasonable. Future research should include direct educational approaches involving team education rather than print-materials only.

## Conclusion and perspectives

Prospectively, GP practices will be increasingly confronted with dementia in patients with migration background. Therefore, our dementia care toolbox is a promising approach to facilitate and potentially improve dementia care especially for patients with migration background. However, further studies are needed to investigate the effectiveness of the dementia care toolbox in a larger sample of GP practices.

## Data Availability

The datasets used and/or analysed for the current study are available from the corresponding author on reasonable request and with permission of the responsible ethics´ committee.

## Abbreviations

CI: Confidence interval
CRT: Cluster-randomised trial
GEE: Generalized estimating eq.
GP: General practitioner
GPACS-D: General practitioner attitudes and confidence scale for dementia
OR: Odds ratio
PrA: Practice assistant
SD: Standard deviation
